# Protection by exclusion? The (lack of) inclusion of adults who lack capacity to consent to research in clinical trials in the UK

**DOI:** 10.1186/s13063-019-3603-1

**Published:** 2019-08-05

**Authors:** Victoria Shepherd, Fiona Wood, Richard Griffith, Mark Sheehan, Kerenza Hood

**Affiliations:** 1 Division of Population Medicine, Cardiff University, Heath Park, Cardiff, CF14 4YS UK; 2 Centre for Trials Research, Cardiff University, Neuadd Meirionnydd, Heath Park, Cardiff, CF14 4YS UK; 3 College of Human and Health Studies, Swansea University, Singleton Park, Swansea, SA2 8PP UK; 4 Ethox Centre, University of Oxford, Big Data Institute, Old Road Campus, Oxford, OX3 7LF UK

**Keywords:** Informed consent, Mental capacity, Proxy, Randomised controlled trials, Inclusion

## Abstract

**Background:**

Around two million adults in the UK have significantly impaired decision-making capacity. However, there are concerns that this population is under-represented in research, due in part to the challenges around obtaining consent. Under-representation of populations denies those who would have wanted to participate the opportunity to make a contribution to society, but also fails to generate results that are applicable to them. Consequently, the evidence base for their care is poorer than for other populations. We recently published in this journal an analysis of Participant Information Sheets provided to consultees and legal representatives of adults who lack capacity and noted the small number of trials designed to include adults who lack capacity. In order to understand how many adults who lack capacity to consent are actually enrolled in clinical trials, we further explored how many of the participants lacked capacity, and who acted as a consultee or legal representative on their behalf.

**Main text:**

The ISRCTN registry was searched for UK clinical trials in conditions commonly associated with cognitive impairment that were designed to include (or not exclude) adults who lack capacity to consent. Details about participants and capacity status were obtained from published data or directly from the trial teams. Of the 80 retrieved clinical trials that had completed in the previous 3 years, we identified 15 which included adults who lack capacity to consent. Data regarding participants’ capacity status were not available for five trials. Where capacity was reported, 5–100% participants lacked capacity to consent. Trials predominantly utilised personal consultees/legal representatives; however, 39% (634/1631) of participants required a professional to act as consultee/legal representative.

**Conclusions:**

Only a small number of trials including adults who lacked capacity were identified. The majority of participants were represented by a personal consultee/legal representative; however, between 21 and 100% of participants across five trials required the involvement of a professional, suggesting it is not uncommon. Data relating to capacity status were rarely reported, potentially masking the under-representation of adults who lack capacity. The findings may help researchers and funders target resources towards studies involving under-represented populations to increase the much-needed evidence base for their care and treatment.

## Background

An estimated two million people in England and Wales have significantly impaired decision-making through conditions such as dementia, mental illness, learning disabilities, or other conditions that affect cognitive function such as delirium or head injury [1]. Adults considered to be unable to make a particular decision or take a particular action for themselves at the time the decision or action needs to be taken, due to an impairment or disturbance in the functioning of the mind or brain, are described as lacking decision-making capacity (Part 1 (2(1, 2) of [2]). This lack of decision-making capacity may be temporary or permanent, and in England and Wales is determined following an assessment process laid down in the Mental Capacity Act 2005 [3]. Up to half of patients in acute medical and psychiatric healthcare settings lack decision-making capacity [4, 5], rising to around 70% in settings such as care homes [6] and approaching 90% in intensive care settings [7]. These populations often have significant co-morbidities [8] and experience the greatest and most complex care needs [9]; therefore, research into conditions that affect these populations is essential in order to improve their evidence-based care. In many population groups there are significant differences between those with and without capacity. For example, older people living in care homes who lack capacity are likely to be more frail than those with capacity and have an increased vulnerability to infection [6]. However, when it comes to clinical trials, considered to provide the best quality evidence [10], groups such as frail older people are often excluded despite bearing a disproportionate burden of disease and medication use [11]. We have previously described the result of having under-researched populations as being evidence *biased* medicine [12].

There is growing recognition of the importance that populations included in clinical trials should adequately represent the population treated in clinical practice [13]. Older people can exhibit unpredictable treatment responses [14, 15], often experience multiple comorbidities, and are more likely to experience adverse drug reactions, yet older people are poorly represented in clinical trials of drugs they are likely to receive [13, 16]. Similarly, people with intellectual disabilities have few data available to inform their pharmacological care [17]. Pharmacokinetic studies rarely address alternative delivery routes such as gastrostomy tubes, and medications are often prescribed for people with intellectual disabilities, especially psychotropic drugs with significant adverse effects, with minimal evidence to support their use [17]. This is due in part to the tendency to exclude adults who lack capacity from clinical trials [18, 19].

Exclusion from research can result in a lack of evidence-based care for such populations, who may already experience significant health disparities [12], resulting in them living in a ‘knowledge shadow’ [20]. Moreover, the assumption that randomised controlled trials have strong external validity can be questioned when certain groups are systematically excluded from those trials [21]. The under-representation of groups, such as those who lack decision-making capacity, in clinical trials has been recognised as a concern by organisations such the UK’s National Institute for Health Research (NIHR), who are seeking to identify under-represented groups and develop innovations in clinical trial design and delivery which could increase recruitment of those groups [22]. Identifying the best approaches to ensure the inclusion and participation of under-represented or vulnerable groups in randomised trials has been recognised as a priority area [23]. Similarly, in the US, the National Institutes of Health (NIH) are actively seeking ways of addressing underrepresentation, attempting to shift regulation towards protecting such groups *through* research, rather than *from* research [17]. This approach is reflected in new ethical guidance which proposes a change to the position that adults lacking capacity to consent to the research *should only be included* if the research is directly relevant to them, to the position that they must be included unless there is a scientific *justification for their exclusion* [24]*.* One of the remaining challenges is identifying which groups are under-represented in research. There is no empirical evidence regarding the amount of research currently being conducted with populations such as those who lack capacity to consent [25].

A small number of studies have reviewed the inclusion and exclusion of specific populations with impaired capacity in clinical trials, such as adults with intellectual disabilities [21], and people with cognitive impairment and dementia [26]. However, many of the challenges around including adults who lack capacity are systemic, such as the complex legal and ethical frameworks [12], or structural, such as requiring access through gatekeepers [27, 28]. Therefore, understanding the number of trials that are designed to include participants who lack capacity, and the proportion of people who lack capacity actually participating, will provide much needed data about the opportunities to participate in research that are available to these populations.

In our study recently published in *Trials*, in which we conducted a content analysis of Participant Information Sheets provided to consultees and legal representatives of adults who lack capacity, we noted the small number of trials designed to include participants who lack capacity [29]. What was missing from this account was an understanding of not just how many trials are designed to include adults who lack capacity to consent, but how many participants who lack capacity are subsequently enrolled in these trials. Knowing how many of the participants enrolled in trials who lacked capacity to consent will aid understanding of the generalisability of the findings to these populations; help identify under-represented or underserved groups; and ensure transparency around the recruitment of under-represented groups. The inadequate recruitment of traditionally under-represented populations prevents researchers from creating tailored interventions [30]; therefore, understanding the populations included in research is an important first step towards eliminating the existing health disparities that in part arise from these research inequalities [31]. We explored this through a further analysis of participant data from trials completed within the preceding three years, which we now describe in this commentary.

We also examined the data to determine, for those participants who lacked capacity to consent, who provided consent or agreement on the participant’s behalf. In England and Wales, the legal frameworks govern the inclusion of adults who lack capacity in clinical trials of medicinal products under the Medicines for Human Use (Clinical Trials) Regulations 2004 [32], with other types of research governed by the Mental Capacity Act 2005 [3]. Under this legal framework, someone who knows the person who lacks capacity is approached by the researcher to act as their legal representative or consultee. In circumstances where no-one is available or willing to act in a personal capacity, then a professional who cares for the person can act as a professional legal representative (usually the doctor primarily responsible for their medical treatment) or nominated consultee on their behalf [3, 32]. Both personal and professional legal representatives are required to provide informed consent on behalf of the adult who lacks capacity based on what they would have wanted had they the capacity to choose for themselves, their ‘presumed will’ [32]. A nominated consultee provides advice regarding trial participation on the same basis as a personal consultee—what the person’s wishes and feelings would be likely to be about taking part in the project if he or she had capacity [3]. This legal basis is regardless of how well the professional legal representative or nominated consultee knows the person who lacks capacity to consent, and so in turn the extent to which they can determine the wishes and feelings of the person. Our previous study identified particular issues with the information provided to professionals acting as a proxy decision-maker (legal representative or consultee). We were therefore interested in examining the data reporting on the use of personal versus professional legal representatives and consultees in order to consider for the first time the extent of the use of professionals as proxy decision-makers in the UK.

Our previous analysis of Participant Information Sheets and this additional study form part of a larger project exploring research involving adults who lack capacity to consent, and the involvement of consultees and legal representatives. This larger project also included a survey of health and social care professionals’ knowledge and understanding about the legal frameworks [33], a qualitative study exploring families’ experiences of acting as consultee or legal representative (DECISION Study; in press), and the development of an intervention to support family members involved in making decisions about research. The study reported in this commentary aimed to identify (1) the number of trials completed in the UK within the last three years that included adults who lack decision-making capacity; (2) the number of participants enrolled in the trials and the percentage who lacked decision-making capacity; and (3) who acted as their proxy decision maker.

### Main text

A search of clinical trials was conducted using the ISRCTN registry [34] to identify trials that included adults who lacked capacity to consent that were registered in the UK. We amended the search strategy that was reported in our *Trials* paper to identify only trials that had been completed in the previous three years. This timeframe was selected in order to reflect current legal frameworks and guidance and allow time to have elapsed for analysis of trial data whilst ensuring that investigators’ contact details were recent enough to enable effective contact. As in our previous study, eligible studies were those that (1) included (or did not exclude) adults who lacked capacity to consent and therefore required proxy (consultee or legal representative) involvement, and (2) had recruited participants in the UK. As the focus of the larger project is on the involvement of consultees and legal representatives we excluded trials that used a deferred consent model or consent waiver in emergency research settings, such as post cardiac arrest, where obtaining consent from a legal representative or consulting others is not reasonably practicable [35].

**Fig. 1:**
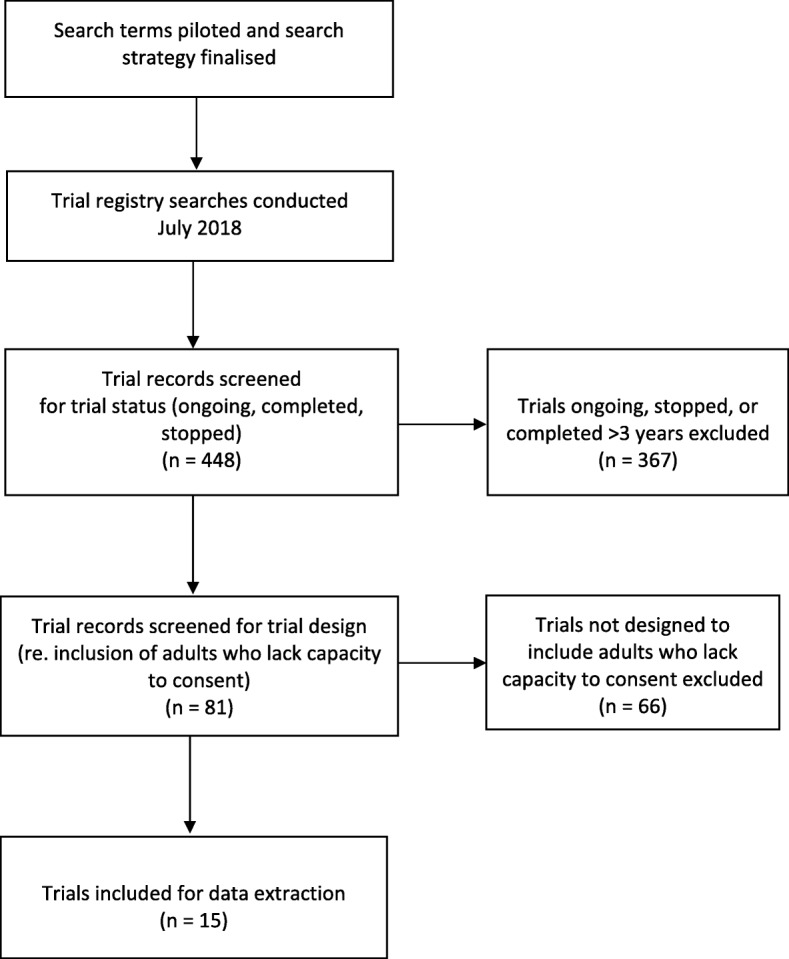
Trial search and data extraction flow diagram

As described in our previous study, trial registries such as ISRCTN are not necessarily intended for searches of this nature [29]. However, they are the only available source for identifying studies across multiple sites, funders, settings, and conditions or populations under investigation. This necessitated a pragmatic search strategy which identified condition or population-specific search terms that would capture trials likely to include adults who lack capacity to consent. The process for trial registry searches, eligibility screening of trials, and data extraction is shown in Fig. 1. The sampling methods used in the published study and in this analysis are derived from similar studies assessing the inclusion of older people [19, 36] and people with intellectual disabilities [21] in medical research.

Searches were conducted in July 2018. Searches were only limited by relevant fields: trial status (completed only), countries of recruitment (United Kingdom), and either condition (e.g. dementia) or text search (e.g. critical care). Trial records were then screened for eligibility by reviewing the participant inclusion and exclusion criteria detailed in the registry record and, where available, the protocol or other study publication. The published participant data were extracted from study publications and reviewed, and the total number of participants, the number of participants who lacked capacity, and details of consultee or legal representative involvement were extracted. Where these data were not available (e.g. where the number or proportion of participants who lacked capacity was not stated or the results were unpublished) the data were requested from the research team, the corresponding authors of study publications, or the Chief Investigator as applicable. As many data were unpublished, trial teams or Chief Investigators were advised when we requested the data that the trials would not be identifiable in our study. Descriptive statistics were used to report the number of studies, total number of participants, and number of participants who lacked capacity. Where known, the number of participants who had a personal consultee or legal representative, versus a nominated consultee or professional legal representative, were reported.

We identified 81 trials in the conditions or populations searched for which were completed between 2015 and 2018, of which 15 trials (18%) allowed for the inclusion of adults who lacked capacity (Table 1).

**Table 1:** Number of trials identified and the number designed to include adults who lack capacity to consent

Trial condition/population	Number of clinical trials completed in previous 3 years	Number of clinical trials including adults who lack capacity to consent completed in previous 3 years
Parkinson’s disease	12	1
Huntington’s disease	2	0
Dementia	25	6
Intellectual disabilities	1	0
Learning disabilities	4	2
Down’s syndrome	0	0
Stroke	25	2
Traumatic brain injury	0	0
Critical care	8	3
Emergency	4	1
TOTAL	81	15

No data were obtained for three trials as there were no publications associated with the trial, and there was no response to our request from the trial team or Chief Investigator. Of the trials where data were available, the number of participants recruited ranged from 22–1148 (Table 2). Where data were available, the proportion of participants who lack capacity ranged from 5 to 100%.

**Table 2:** Characteristics of included trials and number of participants

Study ID	Trial condition/ population	Trial setting	Total participants n (%)	Participants who lacked capacity to consent n (%)	Personal consultee/legal representative n (%)	Nominated consultee or professional legal representative n (%)
01	Parkinson’s disease	Community	76	4 (5%)	4 (100%)	0
02	Dementia	Care homes	34	34 (100%)	34 (100%)	0
03	Dementia	Care homes	40	40 (100%)	0	40 (100%)
04	Dementia	Community	63	23 (37%)	23 (100%)	0
05	Dementia	Hospitals and care homes	14^#^	-	-	-
06	Dementia	Hospitals and care homes	265	-	-	-
07	Dementia	Care homes	987	784 (79%)	336 (43%)	448 (57%)
08	Learning disabilities	Community	312	185^$^(59%)	–	–
09	Learning disabilities	Community	22	18 (82%)	12 (67%)	6 (33%)
10	Stroke	Hospitals and community	345	88 (26%)	88 (100%)	0
11	Stroke	Hospitals	1148*	540*(47%)	425*(79%)	115*(21%)
12	Critical care	Hospitals	120^#^	-	-	-
13	Critical care	Hospitals	103	100 (97%)	75 (75%)	25 (25%)
14	Critical care	Hospitals	84	-	-	-
15	Emergency	Hospitals	81^#^	-	-	-

* Data not verified by investigator

- Data item not available

^$^ Number of participants with a severe or profound learning disability as trial data reported by type of learning disability and not capacity status

^#^ No trial data available, number of participants is planned sample size as stated in protocol

Where data were available relating to the designation of the consultee or legal representative who was involved (*n* = 9), 44.5% (*n* = 4) of trials involved only personal consultees/legal representatives, 11% (*n* = 1) of trials involved only professionals acting as consultee/legal representative, and 44.5% (*n* = 4) had a combination of both personal and professional consultees/legal representative. Trials that used a combination were conducted across a range of settings, including long-term care settings such as care homes (Study ID 07), primary care (Study ID 09), as well as acute critical care settings (Study ID 13). Where data were provided about participants’ consultee or legal representative (*n* = 1631), 39% (*n* = 634) required a professional to act on their behalf.

## Conclusions

Only a small proportion of clinical trials include adults who lack capacity to consent, even in populations with conditions which can be characterised by impaired decision-making capacity. Of trials designed to specifically include adults who lacked capacity, for some trials only around 5% of recruited participants lacked capacity to consent. Our findings are consistent with previous studies which surveyed the inclusion of persons with intellectual disabilities in research and found that only 2% of 300 randomly chosen clinical trials published in high impact medical journals clearly included people with intellectual disabilities [21].

Despite the size of the population of people who lack capacity in England and Wales being roughly equivalent to the number of people living with cancer in the UK [37], the number of clinical trials that include participants who lack capacity appears to be considerably lower than the number of cancer trials. The number of cancer patients in the UK participating in clinical studies has risen dramatically in the past decade from one in 26, to around one in six patients diagnosed [38]. However, despite initiatives such as the UK’s Dementia 2020 Challenge which sought to increase the numbers of people with dementia participating in research [39], the number of people with conditions associated with impaired decision-making capacity, such as dementia, remains low [40]. Our findings suggest that this under-representation is seen in a range of different populations experiencing impaired capacity.

The data suggest that professionals acting as a nominated consultee or professional legal representative for an adult who lacks capacity to consent is not a rare occurrence and, in some trials, they alone take on this role. There may be a number of factors affecting who is involved in decisions about research participation. It may be linked to the timeframes within which the participant needs to be recruited in an acute setting and the subsequent availability of a family member within that timeframe. It was also reported by one trial that family members of care home residents sometimes felt unable to make a decision on behalf of their relative, and therefore consider the care home staff better placed to do so, whilst others failed to respond to contact by the research team. Whilst having legal provisions for professionals to act as consultee/legal representative is important for those who would otherwise be unrepresented, there is currently no advice or guidance available about the role. There are concerns about whether those acting as nominated consultees and professional legal representatives are sufficiently informed and prepared for their role. Our previous research has shown that there is a lack of knowledge amongst health and social care professionals about the legislation governing research involving adults who lack capacity [33], leading to concerns about the confidence and competence of care professionals when including those who lack capacity in their care in medical research. As a result, guidance for professionals is urgently needed beyond that briefly included in the Mental Capacity Act Code of Practice [2]. There is also a need for further research to examine the informational needs of health and social care professionals acting as nominated consultees and professional legal representatives and explore how they approach decision-making in such ethically complex roles.

As reported in our previous study [29] there are a number of limitations to note. Whilst capacity is considered to be time and decision-specific rather than global [3], the circumstances under which a consultee or legal representative is required, and whether their relationship with the adult who lacks capacity to consent is a personal or professional relationship, are relevant factors in this context. It is recognised that a lack of capacity cannot be established by reference to a condition [3], and only some individuals living with these conditions or in these populations will experience any cognitive impairment, or lack capacity to consent to a trial. The searches were limited by the ability to search the registry by condition/subject area or key words only; for example, it was not possible to search by capacity status as an inclusion or exclusion criterion, and only conditions considered to be most likely to include adults with impaired capacity were included in the search. The sensitivity and specificity of search terms were highly variable. Terms such as ‘critical care’ and ‘emergency’ appeared relatively precise; however, ‘care home’ as a text search did not return many trials despite a growing number of trials in care homes [41], and ‘trauma’ as a condition included psychological trauma. Therefore, a pragmatic search strategy was used. The searches cannot be considered to be comprehensive, and eligible trials may have been conducted that are not included in this study. Additionally, although there is an expectation that clinical trials are prospectively registered [42], trials may have been conducted but not registered. Research studies that are not defined as clinical trials are not registered and therefore are not included in this study. Data were not available on the number of participants who lacked capacity, or who acted as consultee or legal representative, for a number of the trials.

We have taken the first step towards understanding the state of play regarding the inclusion of adults who lack capacity to consent in clinical trials in the UK, and who acts as a consultee or legal representative on their behalf. We identified that, firstly, few trials completed in the UK were designed to include adults who lack decision-making capacity, which limits the opportunities for those with cognitive impairments to contribute to research and may impact on the generalisability of the results. Secondly, the overall number of participants enrolled in the trials who lacked decision-making capacity was low, which may contribute to the low evidence-base available for these populations. Lastly, a high proportion of participants required a professional to act as their proxy decision maker, which has received little attention from professional bodies, employing organisations, or those responsible for research governance and policymaking.

The data presented in our published study and further described in this commentary are not surprising, but do provide the first empirical account of the current under-representation of adults who lack capacity to consent in clinical trials. However, empirical data on the number of adults who lack capacity participating in trials remains low. We encourage investigators to report the proportion of participants who lack capacity in order to allow a greater understanding of the representativeness of the trial population, and therefore the applicability of trial results, to the whole clinical population. We have also highlighted the need for guidance and support for health and social care professionals acting as nominated consultees and professional legal representatives. Together with other findings from our project exploring the involvement of consultees and legal representatives in research, we hope our results help researchers, funders, and policy makers target resources towards studies involving under-researched populations and increase the much-needed evidence base for their care and treatment.

## Data Availability

The datasets used during the current study are available from the corresponding author on reasonable request.

## Abbreviations

NIH: National Institutes for Health
NIHR: National Institute for Health Research
